# Relationship between Chewing Ability and Nutritional Status in Japanese Older Adults: A Cross-Sectional Study

**DOI:** 10.3390/ijerph18031216

**Published:** 2021-01-29

**Authors:** Keiko Motokawa, Yurie Mikami, Maki Shirobe, Ayako Edahiro, Yuki Ohara, Masanori Iwasaki, Yutaka Watanabe, Hisashi Kawai, Takeshi Kera, Shuichi Obuchi, Yoshinori Fujiwara, Kazushige Ihara, Hirohiko Hirano

**Affiliations:** 1 Tokyo Metropolitan Institute of Gerontology, Tokyo 173-0015, Japan; ega0dm@gmail.com (Y.M.); mashirobe@gmail.com (M.S.); aedahiro514@gmail.com (A.E.); yohara@tmig.or.jp (Y.O.); iwasaki@tmig.or.jp (M.I.); hkawai@tmig.or.jp (H.K.); obuchipc@tmig.or.jp (S.O.); fujiwayo@tmig.or.jp (Y.F.); h-hiro@gd5.so-net.ne.jp (H.H.); 2 Gerodontology, Department of Oral Health Science, Faculty of Dental Medicine, Hokkaido University, Hokkaido 060-8586, Japan; ywata@den.hokudai.ac.jp; 3 Department of Physiotherapy, Takasaki University of Health and Welfare, Gunma 373-0033, Japan; kera@takasaki-u.ac.jp; 4 Department of Social Medicine, Hirosaki University Graduate School of Medicine, Hirosaki University, Aomori 030-8560, Japan; ihara@hirosaki-u.ac.jp

**Keywords:** nutrient intake, food groups, chewing ability

## Abstract

Objectives: This study aimed to determine the relationship between objective chewing ability and the nutritional status of Japanese community-dwelling elders. Design: A cross-sectional study. Participants: A total of 509 community-dwelling elders living in the Tokyo metropolitan area participated in a comprehensive survey conducted in October 2013. Measurements: The basic characteristics were sex, age, and body mass index. Undernutrition was examined through serum albumin levels. Chewing ability was examined through color-changeable xylitol gum by evaluating the color changes in chewing gum. Nutritional intake was examined using the semi-quantitative food frequency questionnaire. Results: In the poor chewing ability group, all nutrient intake levels were significantly low, except for carbohydrates, and intake levels for all food groups were significantly low, except for cereals, confectionery, sugars, seasonings, and spices. Additionally, after adjusting for covariates for sex, age, Tokyo Metropolitan Institute of Gerontology-Index of Competence (TMIG-IC) score, Mini-Mental State Examination (MMSE) score, body mass index (BMI), stroke, number of functional teeth, energy intake, and protein intake, chewing ability was found to be significantly associated with undernutrition. Conclusion: We concluded that chewing ability was closely associated with nutrient and different food groups’ intake, as well as undernutrition, among Japanese community-dwelling elders. Thus, to ensure comprehensive nutritional management, nutritionists and dentists should collaborate when treating the same patients.

## 1. Introduction

Japan is experiencing unprecedented population aging, transforming the country into a super-aged society. By 2015, the proportion of the population over 65 years old had reached 26.0% [1]. Thus, developed countries with an aging population, such as Japan, urgently need to take measures to provide appropriate care that meets the needs of older adults, who incur increased illness incidences owing to their advanced age. A report from Health Japan 21 reports that about 18% of community-dwelling older adults are undernourished (as per body mass index (BMI)). Additionally, this ratio is expected to increase in the future [2]. Moreover, especially among older adults, undernutrition is related to reduced cure rates, increased risk of complications, and death [3,4,5]. Thus, there is an urgent need to promote early interventions aimed at improving the eating habits of the Japanese older adults to ensure that they have well-balanced meals.

Recently, the term oral frailty has been used to identify people that require early-stage interventions to diminish this frailty. People classified as orally frail need to have a low rating on more than three of the following six oral statuses: The number of natural teeth, chewing ability, articulatory oral motor skill, tongue pressure, subjective difficulties in eating, and swallowing [6].

Oral frailty has been reported to increase the risk of requiring long-term care, sarcopenia, death, and physical frailty [6]. Moreover, decreased chewing ability has been associated with deteriorated life functions, being housebound, bedridden, and the onset of sarcopenia in community-dwelling older women [7]. Chewing is a process that includes predation, crushing and mixing of food, the formation of a bolus, and delivery to the pharynx, which greatly impact food intake [8]. It can be observed for a comprehensive evaluation of oral function [9].

Additionally, impaired dentition among older adults has been reported to be significantly associated with nutritional intake and the intake of different food groups; for example, individuals with impaired dentition have reduced protein, sodium, potassium, calcium, vitamin A, vitamin E, and dietary fiber intake levels compared to those without impaired dentition [10]. Zhu et al. [11] analyzed data from the American National Health and Nutrition Examination Survey (NHANES), which was conducted among nearly 9000 individuals (25% of whom were over 65 years old); after adjusting for socioeconomic factors (i.e., age, sex, race, income, physical activity, and smoking status), the authors established that people with 20 teeth or less had a lower intake of fruits and vegetables compared to people with more than 28 teeth, as well as a lower nutrient intake with missing minerals, proteins, dietary fibers, and most vitamins. Thus, ensuring that people maintain their teeth is important for promoting food intake. However, at present, it is not possible to directly intervene in this matter to enhance the related outcomes. Furthermore, these studies [10,11] did not investigate other possible influencing factors (e.g., undernutrition) and the condition of functional teeth (i.e., the presence or absence of dentures).

Therefore, the purpose of this study was to focus on chewing ability and to cross-sectionally investigate the relationship between objectively evaluated masticatory ability and the nutritional status of the community-dwelling older adults in Japan.

## 2. Materials and Methods

### 2.1. Participants

**Figure 1:**
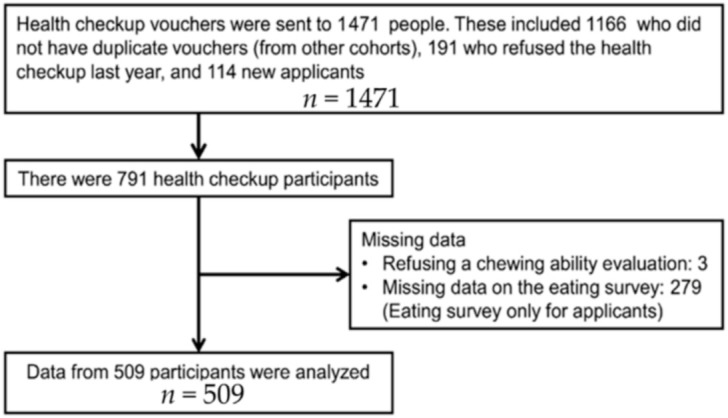
Recruitment of research participants.

The survey was conducted in October 2013 among community-dwelling elders in the urban areas of Tokyo, Japan. Figure 1 delineates recruitment details. Among the 1471 people invited, 791 agreed to participate and were included in the study. Of these, 509 participants (294 women, 57.8%) were analyzed; three that missed the chewing ability required for the evaluation were excluded along with the 279 who did not participate in the eating survey.

### 2.2. Survey Items

#### 2.2.1. Basic Information

A nurse or a trained researcher verbally asked each participant about their gender, age, and instrumental activities of daily living (IADL, as assessed by the Tokyo Metropolitan Institute of Gerontology-Index of Competence: TMIG-IC) [12], cognitive function (Mini-Mental State Examination (MMSE)) [13], and their medical history (high blood pressure, stroke, heart disease, diabetes, hyperlipidemia). Moreover, a dentist or dental hygienist conducted a check for each participant’s number of functional teeth, including dentures.

#### 2.2.2. Evaluation of Chewing Ability

To examine the participants’ chewing ability, we used color-changeable chewing gum (i.e., a gum used to evaluate masticatory performance, Xylitol; Lotte, Saitama, Japan). This procedure involved asking each participant to chew the gum for 1 min; afterward, participants were supposed to spit the gum into a pouch. To evaluate chewing ability, we used a color chart that was divided into five levels [14]: The chewing gum was green, but turned red when chewed; if the color of the gum was close to green, the chewing ability was evaluated as low. The median was the cutoff. In this study, levels 1, 2, and 3 were classified as “poor”, and levels 4 and 5 were classified as “good”.

#### 2.2.3. Body Measurements

We measured using a height scale the height (cm) and weight (kg) and calculated their BMI.

#### 2.2.4. Undernutrition Diagnosis

To examine undernutrition, participants had their blood drawn and were subjected to evaluation—using blood serum albumin values. For this analysis, albumin (4.0 g/dL) was designated as the cutoff for classifying participants into two groups: Undernutrition and healthy groups [15].

### 2.3. Eating Survey

An eating survey was administered to consenting participants. To evaluate participants’ nutrient and food group intake, we used the semi-quantitative Food Frequency Questionnaire (FFQ), which was developed by Takahashi et al. [16]. This questionnaire was based on 29 food groups and was conducted by a nutritionist. Participants were asked about their food intake over the past month, food portion sizes, and the amount of food consumed in one meal. The residual correction method was used with energy intake from nutrient and food group intake figures [17]. The dietary survey was conducted by a trained and registered dietitian who standardized the listening method.

### 2.4. Statistical Analysis

We conducted comparative analyses for chewing ability using an independent *t*-test for continuous variables and a chi-squared test for categorical variables. Furthermore, to calculate the deficiency ratio of the poor chewing group, we used the ratio of the good chewing group as the standard; then, we compared the participants’ differences in nutrient and food group intake by chewing ability (intake in poor chewing group/intake in good chewing group × 100). Additionally, we examined the relationship between chewing ability and undernutrition, adjusting for BMI and the number of functional teeth. Finally, to analyze the relationship between undernutrition and chewing ability, we used logistic regression analysis. Statistical analyses were conducted using SPSS 25.0 (IBM, Tokyo, Japan) and significance was set at less than 5%.

### 2.5. Ethical Considerations

This study was approved by the Ethics Committee of the Tokyo Metropolitan Geriatric Hospital and the Institute of Gerontology (approval No. 2013, 1253). Before participation, all participants and their legal representatives (e.g., family members) were provided with an oral/written explanation of the study objectives, methods, and expected outcomes, after which they provided informed consent. We affirm that we have taken all measures to ensure data anonymity and masking.

## 3. Results

Participant characteristics based on chewing ability are displayed in Table 1. In total, 301 participants (59.1%) had poor chewing ability and 208 had good chewing ability (40.9%). Moreover, 60 participants (11.8%) were undernourished, whereas 449 (88.2%) had good nutritional status.

**Figure 2:**
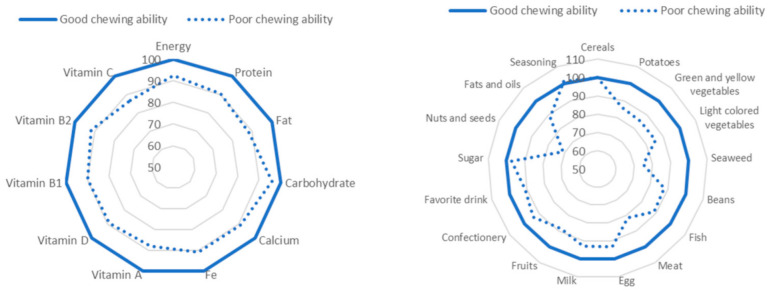
The difference between poor and good chewing ability when the good chewing ability is set to 100.

Table 2 shows the participants’ nutrient and food group intake based on chewing ability. In the poor chewing ability group, energy, protein, fat, calcium, iron, vitamin A, vitamin D, vitamin B1, vitamin B2, and vitamin C had intake levels that were significantly low, except for carbohydrates, thus including all food groups. Furthermore, the poor chewing ability group had intake levels that were significantly low in contrast, such as potatoes, vegetables, seaweed, beans, fish, meat, eggs, milk, fruits, usual drinks (such as coffee, tea, soft drinks, and alcohol), nuts, seeds, and fats and oils. Figure 2 presents a comparison of food group intake with nutrient intake level according to masticatory function. If the good group is designated a score of 100, then in the poor group, nutrients with a value over 10% are protein, fats, iron, vitamin A, and vitamin C. Food groups with a value over 10% are potatoes, yellow vegetables, seaweed, beans, seafood, meat, and nuts.

**Figure 3:**
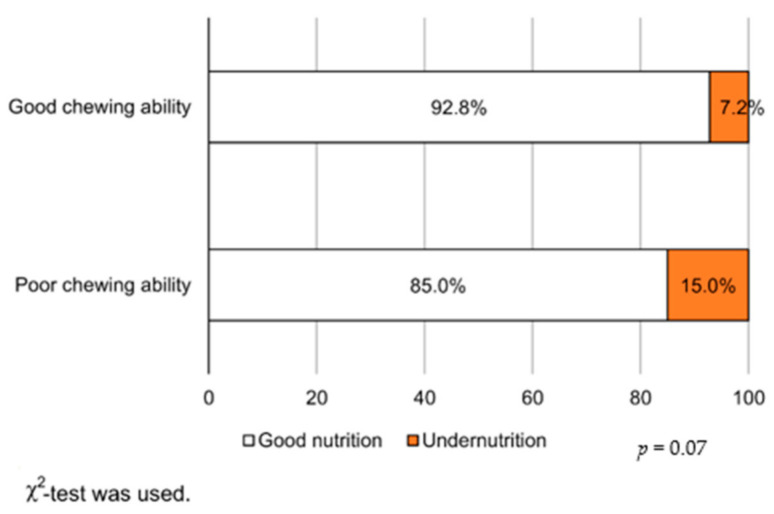
Rates of undernutrition due to differences in chewing ability.

Figure 3 presents the association between chewing ability and undernutrition. In the poor chewing ability group, the undernutrition percentages were higher. There was also a significant association between the gum color chart and albumin levels (Supplemental Data Table S1). There was no significant difference in the association between albumin levels and functional teeth (Supplemental Data Figure S2).

A logistic regression analysis regarding the relationship between chewing ability and undernutrition (Table 3) showed that even when controlling for sex, age, TMIG-IC score, MMSE score, BMI, stroke, number of functional teeth, energy intake, and protein intake, chewing ability was found to be independently and significantly correlated with undernutrition (OR, 1.453; 95% CI, 1.004–2.022; *p* = 0.027).

## 4. Discussion

Our results showed that chewing ability was related to nutrient and food group intake among community-dwelling older adults in urban areas of Japan. Moreover, after adjusting for BMI and the number of functional teeth, the chewing ability was significantly related to undernutrition. Thus, good chewing ability among older adults may help them maintain a high dietary variety; in turn, this can lead to the maintenance of nutritional status. As far as we know, this study is novel in that it examines the relationship between the objectively evaluated chewing ability, nutritional intake, and nutritional status of older adults living in Japan.

In the current study, chewing ability was associated with nutrient and food group intake and undernutrition, even when adjusting for the number of functional teeth, including dentures. Previous studies have shown that people with impaired dentition have substantially lower protein, sodium, potassium, vitamin A, vitamin E, and dietary fiber intake compared to those without it [10]. Additionally, as mentioned in the Introduction section, Zhu et al. [11] analyzed data from 9000 American people and found that those with 20 teeth or less had lower fruit and vegetable intake compared to those with 28 teeth or more; even after adjusting for socioeconomic factors (e.g., age, gender, race, and income), physical activity, and smoking, they had a lower intake of minerals, proteins, dietary fibers, and most vitamins. Nonetheless, although these studies have illustrated the importance of maintaining an appropriate number of teeth for food intake, interventions regarding the number of teeth, in itself, are currently not possible. Moreover, previous studies failed to investigate oral function and the presence or absence of dentures. In this study, we also investigated functional teeth.

Conversely, Inomata et al. [18] compared people’s number of teeth with the occlusal force exerted when chewing and found that occlusal force was related to walking speed and the intake of green-yellow vegetables, seafood, dietary fiber, and vitamins. Furthermore, among the people without teeth (i.e., full dentures), about 60% answered that they could still eat hard foods such as pickled radish or dried squid [19]. These results indicate that even full dentures contribute to the practical recovery of one’s chewing ability. Additionally, according to results from the 2011 Survey of Dental Diseases, tooth retention among Japanese older adults had greatly improved: The percentage of seniors over 80 years old who retained more than 20 teeth was 50% [20]. Conversely, a study showed that the current measures taken to improve oral function, including swallowing, remain inadequate [21]. Therefore, our results seem to support previous studies and show that understanding oral function is important for maintaining and improving nutritional status.

Our results further indicated that, among the poor chewing ability group, all nutrient intake levels were significantly low, except for carbohydrates, and that intake levels for all food groups were significantly low, except for cereals, confectionery, sugars, seasonings, and spices. Among older adults, the protein intake derived from meat and seafood is especially important as a countermeasure to frailty. In old age, although muscle mass and functionality are naturally reduced, studies have shown that a causal factor of such diminishment relates to the anabolic suppression response of skeletal muscle formation [22]; when compared with that during youth, it was shown to be weakened, and the reason may be that aging brings about protein anabolic resistance. Hence, a study showed that when a woman of frail status and aged over 65 years was fed a high-protein diet (1.23 g/kg/day), her protein assimilation increased and her nitrogen equilibrium became positive, suggesting the effectiveness of a high-protein diet for older adults [23].

However, the importance of dietary variety, rather than the intake of single foods or nutrients, has also recently been highlighted. In our previous research, we awarded participants one point to eat each of these ten foods every day [24]: Seafood, meat, eggs, milk, soybeans/soybean products, green-yellow vegetables, seaweed, potatoes, fruits, and fats/oils. Participants could score a maximum of 10 points, and if they did not eat any of these food, no points were awarded. In this study, we used the Kihon-checklist criteria to distinguish between the frail, pre-frail, and robust groups and found that the frail group scored 3.9, the pre-frail 4.3, and the robust 4.5 points, corroborating a previous study reporting a significant relationship between participants’ frailty levels and their dietary variety score, even after adjusting for gender, age, drinking and smoking status, past medical history, blood serum albumin values, and energy intake [25]. Moreover, the dietary variety is associated with not only frailty but also cognitive function, and a longitudinal study reported that high dietary variety reduces the risk of cognitive decline by 44% [26]. Varying food types may lead to an adequate intake of proteins, vitamins, and minerals, which may contribute to the maintenance of muscle mass, muscle strength, and cognitive function. Therefore, feeding variation is an important indicator of nutritional support in older adults. Nonetheless, our results showed that poor chewing ability may lead to the reduced intake of protein (e.g., from meat and fish) and decreased dietary variety, both of which can potentially affect people’s nutritional status, thus possibly leading to undernutrition. To put our results into perspective, our sample comprised Japanese older adults that were relatively healthy, living independently in the community, and that chose to participate in the outpatient-style health check independently. Even in this population, there was an association between poor chewing ability, food intake, and low nutrition, suggesting that earlier support and intervention may be necessary. Thus, to support Japanese older adults’ food intake, practitioners and stakeholders related to their nutritional health should endeavor to understand their oral function and denture status while aiming to provide cooperative care that unites nutrition and dental departments at earlier stages, rather than just offering nutritional guidance/meal support.

Recently, intervention studies have combined nutritional guidance, oral function improvement interventions, and the use of prosthetics; Bradbury et al. [27] divided the study sample between a “complete dentures only” group and a “complete dentures with nutritional guidance” group, and reported that the second group experienced significant improvements in the intake of vegetables and fruits. Moreover, Kikutani et al. [28] divided a sample of people that required long-term care between the “meal support only” group and the “meal support with oral function training” group, showing that the serum albumin values of the second group increased significantly. Furthermore, Suzuki et al. [29] reported that the provision of simple nutritional support amid denture provision is effective in improving patients’ nutritional intake and chewing ability. Thus, previous studies concur with our statement regarding the need for nutrition and dentistry to collaborate, as this collaboration can contribute to maintaining Japanese older adults’ health, which may allow for more positive influences on their healthy life expectancy, and similar outcomes may not be achievable by conducting oral management and nutritional support separately. Finally, we believe that future intervention studies aimed at examining the effects of the collaboration between nutrition and dental departments and identifying the causal relationships between our studied variables are warranted.

Although we provided fruitful results in the literature, this study had several limitations: (1) We used a cross-sectional design; therefore, our study cannot indicate causality. (2) As this survey required participants to go to the venue, our participants were relatively healthy; in particular, the eating survey was conducted by consenting participants, implying that our sample may comprise a population with higher health and dietary literacy. (3) This survey was conducted in one urban area. Therefore, it is unclear whether the same results will be obtained for older adults living in other urban areas or rural areas. Future studies are required to generalize our findings. (4) Albumin was used for the evaluation of undernutrition, and a previous study reported that the evaluation of undernutrition of albumin alone is insufficient. In the future, evaluation using Mini Nutritional Assessment, Malnutrition Universal. Screening Tool, etc. is required [30]. (5) Of the dietary surveys, FFQ has been reported to be reminiscent and biased, and caution is required when interpreting the results [31]. Moreover, the interviews conducted in this study were standardized.

Accordingly, future intervention and longitudinal studies aimed at identifying causality in the relationship between objective chewing ability and nutrition are warranted.

## 5. Conclusions

Our results indicated a significant association between chewing ability with nutrient and food group intake and undernutrition among Japanese older adults. Thus, nutrition management should be conducted with an understanding of and guided by patients’ oral function and denture status; moreover, we highlight that nutritionists and dentists should endeavor to cooperate when treating the same patients, as this can produce better outcomes than separate interventions. In Japan, there is no medical fee service in which dentists and nutritionists cooperate. Collaboration between dentists and nutritionists has now become imperative. Future studies that explore effective approaches to maintain chewing ability and assess whether such approaches have beneficial effects on the nutritional status in older adults would be an interesting next step.

## Figures and Tables

**Table 1:** Subject characteristics compared to chewing ability.

Characteristics		Poor Chewing Ability (*n* = 301)			Good Chewing Ability (*n* = 208)			*p*-Value
Sex	female	60.3%			53.8%			0.171
Age	year	73.9	±	5.8	72.1	±	5.1	<0.001
BMI	kg/m^2^	23	±	3.1	23	±	3.3	0.964
Fat-free Mass Index	kg/m^2^	16.1	±	1.6	16.4	±	2.1	0.113
Serum albumin	g/dL	4.28	±	0.2	4.34	±	0.2	0.001
TMIG-IC	score	12.2	±	1.2	12.3	±	1.3	0.203
MMSE	score	28.3	±	2.0	28.7	±	1.6	0.071
Functional teeth	teeth	26.3	±	4.1	27.6	±	1.8	<0.001
Hypertension	positive	47.4%			41.3%			0.205
Stroke	positive	9.3%			7.2%			0.517
Cardiovascular disease	positive	16.6%			14.4%			0.538
Diabetes	positive	14.6%			11.5%			0.355
Hyperlipidemia	positive	32.8%			34.6%			0.703
Chronic obstructive pulmonary disease	positive	0.7%			1.0%			1.000
Cancer	positive	14.2%			11.5%			0.425
Depression	positive	1.0%			3.4%			0.099

For continuous variables, we used the *t*-test; for categorical variables, we used the χ^2^-test. Values are expressed as a percentage or mean ± standard deviation.

**Table 2:** Nutrient and food groups intake compared to chewing ability.

Nutrient and Food Groups		Poor Chewing Ability (*n* = 301)			Good Chewing Ability (*n* = 208)			*p*-Value
Energy	kcal	1859	±	486	2009	±	599	0.002
Protein	g	66.9	±	15.5	74.0	±	19.5	<0.001
Fat	g	60.6	±	18.9	68.5	±	23.9	<0.001
Carbohydrate	g	248.5	±	59.7	259.0	±	75.2	0.081
Calcium	g	641	±	172	705	±	216	<0.001
Iron	g	7.9	±	2.2	8.7	±	2.8	<0.001
Vitamin A	μg	571	±	141	649	±	178	<0.001
Vitamin D	μg	8.3	±	2.0	9.3	±	2.5	<0.001
Vitamin B1	mg	0.9	±	0.3	1.0	±	0.3	<0.001
Vitamin B2	mg	1.1	±	0.3	1.2	±	0.4	<0.001
Vitamin C	mg	104	±	28	119	±	35	<0.001
Cereals	kcal	601.5	±	68.8	600.4	±	86.6	0.871
Potatoes	kcal	23.9	±	5.7	27.7	±	7.1	<0.001
Green-yellow vegetables	kcal	23.9	±	4.6	28.1	±	5.7	<0.002
Light-colored vegetables	kcal	36.1	±	8.2	42.2	±	10.4	<0.003
Seaweed	kcal	2.1	±	0.5	2.8	±	0.6	<0.004
Pulses	kcal	88.0	±	17.7	100.1	±	22.2	<0.005
Fish and shellfish	kcal	115.0	±	24.8	129.3	±	31.3	<0.006
Meat	kcal	159.1	±	49.4	195.6	±	62.2	<0.007
Eggs	kcal	39.3	±	7.2	42.1	±	9.0	<0.008
Milk products	kcal	156.4	±	34.4	168.4	±	43.3	<0.009
Fruits	kcal	70.6	±	20.7	80.1	±	26.1	<0.010
Confectionery	kcal	266.9	±	154.5	284.4	±	194.6	0.260
Usual drinks	kcal	81.4	±	28.1	89.5	±	35.3	0.004
Sugar and sweeteners	kcal	40.0	±	11.8	41.2	±	14.9	0.330
Nuts and seeds	kcal	14.6	±	5.2	20.6	±	6.6	<0.001
Fats and oils	kcal	90.5	±	21.0	102.4	±	26.5	<0.001
Seasoning	kcal	55.7	±	13.4	54.9	±	16.9	0.560

For continuous variables, we used a *t*-test. Values are expressed as mean ± standard deviation.

**Table 3:** Relationship between chewing ability and nutritional status.

Independent Variable	Odds Ratio	95% CI	*p*-Value
Model 1			
Chewing ability (0: good, 1: poor)	1.550	(1.205–1.994)	<0.001
Model 2			
Chewing ability (0: good, 1: poor)	1.453	(1.004–2.022)	0.027

Dependent variables: Serum albumin values (cut-off: 4.0 mg/dL; 0: Good nutrition, 1: Undernutrition). Model 1 was adjusted for sex and age. Model 2 was adjusted for Model 1 plus the Tokyo Metropolitan Institute of Gerontology-Index of Competence score, Mini-Mental State Examination score, body mass index, stroke, number of functional teeth, energy intake, and protein intake.

## Data Availability

The data presented in this study are available on request from the corresponding author. The data are not publicly available due to ethicolegal restrictions imposed by the Ethics Committee at Tokyo Metropolitan Institute of Gerontology.

## Supplementary Materials

The following are available online at https://www.mdpi.com/1660-4601/18/3/1216/s1.
