# Supplementary material for: Relationship between Chewing Ability and Nutritional Status in Japanese Older Adults: A Cross-Sectional Study
Source: Int J Environ Res Public Health. 2021 Jan 29;18(3):1216. doi: 10.3390/ijerph18031216 (PMC7908427; doi:10.3390/ijerph18031216)
Supplement: Supplementary file 1 [file ijerph-18-01216-s001.pdf]

Supplemental Data

Table S1. Relationship between color-changeable xylitol gum color chart and albumin level (g/dL).

		Albumin level			
**Color Chart**	***n***	**average**		**SD**	***p* for Trend**
1	25	4.26	±	0.25	0.017
2	110	4.26	±	0.27	
3	166	4.29	±	0.22	
4	153	4.35	±	0.23	
5	55	4.35	±	0.25	

We used a trend-test. Values are expressed as mean ± standard deviation

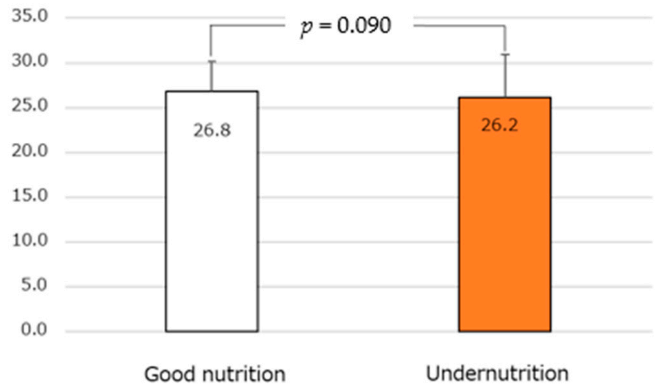

Figure S2. Relationship between nutritional status and functional teeth. We used a t-test. Values are expressed as mean ± standard deviation.
